# The Inhibition of Macrophage Foam Cell Formation by 9-Cis β-Carotene Is Driven by BCMO1 Activity

**DOI:** 10.1371/journal.pone.0115272

**Published:** 2015-01-28

**Authors:** Noa Zolberg Relevy, Sapir Bechor, Ayelet Harari, Ami Ben-Amotz, Yehuda Kamari, Dror Harats, Aviv Shaish

**Affiliations:** 1 The Bert W. Strassburger Lipid Center, Sheba Medical Center, Tel-Hashomer, 5265601, Ramat-Gan, Israel; 2 Sackler Faculty of Medicine, Tel-Aviv University, Tel-Aviv, Israel; 3 N.B.T., Eilat, Israel; Brigham and Women’s Hospital, Harvard Medical School, UNITED STATES

## Abstract

Atherosclerosis is a major cause of morbidity and mortality in developed societies, and begins when activated endothelial cells recruit monocytes and T-cells from the bloodstream into the arterial wall. Macrophages that accumulate cholesterol and other fatty materials are transformed into foam cells. Several epidemiological studies have demonstrated that a diet rich in carotenoids is associated with a reduced risk of heart disease; while previous work in our laboratory has shown that the 9-cis β-carotene rich alga *Dunaliella* inhibits atherogenesis in mice. The effect of 9-cis β-carotene on macrophage foam cell formation has not yet been investigated. In the present work, we sought to study whether the 9-cis β-carotene isomer, isolated from the alga *Dunaliella*, can inhibit macrophage foam cell formation upon its conversion to retinoids. The 9-cis β-carotene and *Dunaliella* lipid extract inhibited foam cell formation in the RAW264.7 cell line, similar to 9-cis retinoic acid. Furthermore, dietary enrichment with the algal powder in mice resulted in carotenoid accumulation in the peritoneal macrophages and in the inhibition of foam cell formation *ex-vivo* and *in-vivo*. We also found that the β-carotene cleavage enzyme β-carotene 15,15’-monooxygenase (BCMO1) is expressed and active in macrophages. Finally, 9-cis β-carotene, as well as the *Dunaliella* extract, activated the nuclear receptor RXR in hepa1-6 cells. These results indicate that dietary carotenoids, such as 9-cis β-carotene, accumulate in macrophages and can be locally cleaved by endogenous BCMO1 to form 9-cis retinoic acid and other retinoids. Subsequently, these retinoids activate the nuclear receptor RXR that, along with additional nuclear receptors, can affect various metabolic pathways, including those involved in foam cell formation and atherosclerosis.

## Introduction

Atherosclerosis is a major cause of morbidity and mortality in developed societies. The disease is characterized by the accumulation of deposits of fatty substances, cholesterol and cellular waste products in the inner linings of large and medium-sized arteries. Inflammatory cells, including monocytes, lymphocytes and macrophages, play important roles throughout the developing stages of atherosclerosis [1–3]. The atherogenic process begins when activated endothelial cells recruit monocytes and T-cells from the bloodstream into the arterial wall. Macrophages that accumulate lipoprotein-derived cholesterol and other fatty materials are transformed into foam cells [2,4]. With time, these fat-laden foam cells increase both in size and number and form deposits in the arterial wall that can ultimately lead to a reduction in blood flow to the brain or to the heart, leading to heart disease [5].

Several epidemiological studies have demonstrated that a diet rich in carotenoids is associated with a reduced risk of heart disease [6–10]. However, the administration of synthetic all-trans β-carotene failed to reduce cardiovascular disease [11]. These negative results observed with the synthetic all-trans β-carotene motivated us to investigate whether other natural isomers of β-carotene, such as 9-cis β-carotene, may play a beneficial role in atherosclerosis.

The 9-cis β-carotene isomer levels are lower than the all-trans isomers in our diet. This isomer is present mainly in fruits and vegetables, with its highest known levels in the unicellular, halo-tolerant alga *Dunaliella bardawil*. When cultivated under appropriate conditions of nitrate starvation and high-light intensity, β-carotene comprises up to 10% of the algal dry weight, and is composed of approximately 50% all-trans and 50% 9-cis β-carotene isomers [12,13]. Due to these properties, we have used *Dunaliella* powder as a rich source of natural β-carotene isomers to examine the effects of 9-cis β-carotene on atherosclerosis and related risk factors. We first demonstrated that a 9-cis-rich β-carotene enriched diet, provided as *Dunaliella* powder, augmented the effects of fibrate on plasma HDL cholesterol and triglyceride (TG) levels in humans, and enhanced the effects of the fibrate on the HDL-cholesterol elevation in human apolipoprotein (apo) AI transgenic mice [14]. In Low Density Lipoprotein Receptor deficient (LDLR-/-) mice, we showed that the 9-cis β-carotene-rich diet inhibited atherogenesis, reduced non-HDL plasma cholesterol levels, and inhibited fatty liver development and inflammation, while the high-dose of synthetic all-trans β-carotene accelerated atherosclerosis [15]. We further found that the 9-cis β-carotene rich diet lowered plasma cholesterol levels and inhibited atherosclerosis progression in high-fat diet fed apoE-/- mice, with established atherosclerotic lesions [16]. Although 9-cis β-carotene reduced plasma cholesterol in these studies, we hypothesized that the conversion of 9-cis β-carotene to retinoids may inhibit atherogenesis by additional mechanisms.

β-carotene is a precursor of retinoids, including retinal, retinol and retinoic acid. All-trans β-carotene is a precursor of all-trans retinoic acid, and 9-cis β-carotene has been shown to be a precursor of all-trans and 9-cis retinoic acid both *in-vitro* and *in-vivo* [17,18]. While both are ligands of the nuclear retinoic acid receptor (RAR), only 9-cis retinoic acid binds to the retinoid X receptor (RXR) [19]. As retinoic acid and other β-carotene metabolites are known to regulate metabolic pathways involved in atherogenesis [20], we presumed that 9-cis β-carotene has the potential to inhibit atherogenesis via its conversion to 9-cis retinoic acid and other metabolites.

The transformation of arterial wall macrophages to foam cells is a key process in the development of atherosclerosis [3]. Very few studies have investigated the effects of carotenoids on foam cell formation, or on the process of reverse cholesterol transport (RCT) from macrophages: the carotene lycopene dose-dependently reduced intracellular total cholesterol in macrophages *in-vitro* [21,22]; while the xanthophyll astaxanthin increased the process of reverse cholesterol transport in macrophages *in-vitro*, however, very high doses were required to attain this inhibitory effect [23]. While both all-trans and 9-cis retinoic acid increased Reverse Cholesterol Transport (RCT), all-trans β-carotene failed to affect the RCT in macrophages *in-vitro* [24]. The outcome of the 9-cis β-carotene administration on macrophage foam cell formation has not yet been investigated. Therefore, we sought to study whether the 9-cis β-carotene isomer isolated from the alga *Dunaliella*, can inhibit macrophage foam cell formation by its conversion to retinoids.

## Materials and Methods

### Mice

Twelve-week-old male LDL receptor knockout (LDLR-/-) mice with C57BL6 genetic backgrounds (Jackson Laboratories) were used in all of the experiments. The mice were housed in plastic cages with a 12 h light/12 h dark cycle and free access to food and water. The study mice were euthanized with isoflurane, and the Animal Care and Use committee of the Sheba Medical Center, Tel-Hashomer, approved all animal protocols (833/13).

### Diets

Two commercial diets were used: a non-purified, low-fat diet (18% protein, 5% fat; TD2018, Harlan Teklad) and a semi-purified high-fat diet (17.3% protein, 21.2% fat, 0.15% cholesterol; TD88137, Harlan Teklad). To enrich the diet with β-carotene, we used powder of the alga *Dunaliella bardawil* containing 6% β-carotene (weight/weight), comprised of 50% all-*trans* and 50% 9-*cis* isomers [13] (kindly provided by Nikken Sohonsha, Japan). In order to prepare the feed, 0.25 L of distilled hot water was mixed with 14 g of gelatin until the solution was clear. Then, 1 kg of powdered feed and *Dunaliella* powder (80 g/kg feed, containing 3 g/Kg all-*trans* β-carotene and 3 g 9-*cis* β-carotene) were thoroughly mixed with the warm gelatin solution. After solidification, the feed was divided into tablets and stored at −20°C in the freezer; the feed was replaced every other day to minimize the oxidation and degradation of its ingredients.

### Study design

Exp.1: Ten, 12-week-old male LDLR-/- mice were allocated into two groups, five animals per group. The control group was fed a regular diet (chow) with no supplementations. The *Dunaliella* group was fed a diet fortified with the algal powder. After 4 weeks of treatment, the mice were injected with thioglycolate (intra-peritoneal) followed by the isolation of peritoneal macrophages.

Exp.2: Ten, 12-week-old male LDLR-/- mice were allocated into two groups, five animals per group. The control group was fed a high fat diet (western) with no supplementations. The *Dunaliella* group was fed a high fat diet fortified with the algal powder. After 6 weeks of treatment, the mice were injected with thioglycolate (intra-peritoneal) followed by the isolation of peritoneal macrophages.

### Peritoneal macrophage production

Mouse peritoneal macrophages were isolated as described previously [25]. These isolated macrophages were counted and seeded at 1.5×10^6^ cells per ml.

### Tissue culture

The cells were grown in DMEM 4.5 g/L glucose containing 10% FCS (Biological Industries Ltd), 50 U/ml penicillin and 50 μg/ml streptomycin. Two cell lines were used: Raw264.7, mouse macrophage cell line, enriched with 2 mM glutamine, purchased from ATCC (TIB-71); and Hepa1-6, mouse hepatoma cell line, enriched with 4 mM glutamine, purchased from ATCC (CRL-1830).

For BCMO1 activity, the cells were seeded in a 100 mm plates, at 6×10^6^ cells per plate. Forty-eight hours after seeding, the cells were treated with β-carotene for 24 hours and analyzed for the presence of retinol.

BCMO1 protein levels were determined by western blot analysis. RAW 264.7 macrophage cells were treated for 24 hours with vehicle (TWEEN 40), 2 μM of 9-cis β-carotene or all-trans β-carotene. The results represent one of five independent experiments.

Retinol, retinal and retinoic acid were dissolved in DMSO with a final concentration of 0.5% DMSO in the cell medium. β-carotene was dissolved in hexane, and the concentration was determined by 450 nm absorbance, followed by the addition of tween40 in acetone (1:4 respectively) to a total concentration of 0.1% tween in the cell medium. Finally, the solvents were evaporated and the residue was solubilized in the medium.

The *Dunaliella* extraction was carried out by dissolving the alga powder in absolute ethanol, with the addition of an identical volume of hexane and 1 mL of DDW. After 30 seconds of vortex spinning, the extract was centrifuged for 5 minutes at 2,000 g, and the upper phase was separated for carotenoid concentration determination.

### Western blot analysis

At the end of incubation with βc, cells were lysed in RIPA buffer (R0278, Sigma, St. Louis, MO, USA). The lysates were incubated on ice for 30 minutes and were centrifuged for 20 minutes at 4°C at 13000 g, and the supernatants were then taken. Protein concentrations were determined by a Pierce BCA Protein Assay Kit (Thermo Scientific). Cell lysates were added with 4X Laemmli sample buffer, and 40 μg proteins were separated on a 7% SDS polyacrylamide gel. Proteins were transferred to nitrocellulose membranes. After non-specific blocking with BSA for 1.5 hours, the membranes were incubated with anti-BCMO1 (Santa Cruz Biotechnology, sc-163736), overnight at 4°C. The membranes were then washed three times with Tris-buffered saline added with 0.1% Tween 20 (TBST), and then incubated with an appropriate HRP-conjugated secondary antibody. Membranes were washed three times with TBST, incubated with an ECL solution (Pierce), and exposed to X-ray films. Bands were quantified by densitometry and normalized to those of β-actin (Santa Cruz).

### LDL isolation and preparation of minimally modified LDL

The LDL was obtained from healthy volunteers by sequential ultracentrifugation (density, 1.063 g/ml) [26], and the concentration was determined by the Lowry method [27]. The Helsinki Committee of the Sheba Medical Center approved all procedures (1340–14), and the research was conducted with full exemption from informed consent. The IRB/ethics committee at Sheba Medical Center specifically waived the need for informed consent Existing plasma samples were pooled and used in the experiments with no identifiers linking individuals to the samples. In order to obtain minimally modified LDL, the LDL was frozen and thawed just before use.

### Foam cell formation *in-vitro* and *ex-vivo*

Foam cell formation was conducted by incubating macrophages (cell line or peritoneal) with 100 μg/ml minimally modified LDL for 24 hours in serum free medium, as previously described [28], along with the relevant treatment of carotenoid or retinoid. For Oil Red O staining, the macrophages were seeded on a 12 mm cover glass in a 12-well plate. Oil Red O staining was done according to Xu et al. [29].

### Transient transfection and luciferase reporter assay

For the RXR luciferase reporter assay, Hepa1-6 cells were transfected with the RXR-Luciferase plasmid (reporter construct containing RXR responsive element) (Qiagene). Hepa1-6 cells were seeded in a 24-well plate, at 200,000 cells per well. Twenty-four hours post seeding, the cells were transfected with the plasmid using JetPEI (Polyplus transfection), and 24 hours post transfection, the cells were treated with the relevant carotenoid/retinoid in a serum free medium for another 24 hours.

The luciferase activity was measured by the Dual Luciferase Reporter Assay kit (Promega) in Fluoroscan Ascent FL (Thermo). The firefly luciferase activity results were normalized to *Renilla* (mediated by CMV promoter).

### Retinol analysis

The cells (~10×10^6^) were scraped and suspended with 1 mL of 10% KOH in absolute ETOH for 20 minutes, in a 55°C water bath for saponification. Following incubation, 2 mL of hexane and 1 mL of DDW were added, and the samples were mixed and centrifuged for 1 minute at 800 g. After centrifugation, the hexane layer was separated and another 0.5 mL hexane was added to the aqueous phase for two more cycles of centrifugation and separation. The hexane layers were dried under a stream of N_2_. The dried samples were suspended in 200 μL methanol, and the retinol concentrations were determined by reverse phase HPLC on a Vydac C18 column (201TP-54, 250 × 5 mm, 5-μm particle size; Vydac, Hesperia, CA) with methanol/butanol/water and 10 mM ammonium acetate as the mobile phase, at a flow rate of 0.8 mL/min [30]. The retinol was detected by monitoring its absorbance at 325 nm, and by comparison with the retention times of the authentic standards. The results are expressed as the nanogram of retinol per 10^6^ cells seeded.

### β-carotene analysis

Mouse plasma (~0.5mL) or peritoneal macrophages were extracted with 2 mL of ethanol containing 10 μM of butylated hydroxytoluene, following the addition of 2 mL hexane and 1 mL of DDW. The samples were mixed and centrifuged for 5 min at 1000 g, and the hexane layer was separated and dried under a stream of N_2_. The dried samples were suspended in 100 μL hexane, and the β-carotene concentrations were determined by reverse phase HPLC on a YMC C30 column (CT995031546QT, 150×4.6, 3μm particle size; YMC Inc., USA) with methanol/methyl-tert-butyl-ether/water with 1.5% ammonium acetate as the mobile phase, at a flow rate of 1 mL/min [31]. The β-carotene was detected by monitoring its absorbance at 450 nm and by comparison with the retention times of the authentic standards. The results are expressed as the nanogram of β-carotene per 10^6^ cells seeded, or as the microgram per 1 mL of plasma.

### Lipid analysis

Fat loaded macrophages were extracted using the Folch method [32]. Briefly, adherent cells were washed with PBS, scraped, mixed with PBS 0.1% triton and incubated for 30 minutes at 37°C. The cell solution was then centrifuged at 12,000 g for 30 minutes; the upper phase was extracted with the Folch solution and incubated for 30 minutes in a 37°C water bath. Afterwards, the lower phase (chloroform) was separated and dried under a stream of N_2_. The residue was solubilized in PBS 1% triton and underwent an enzymatic colorimetric procedure using a total cholesterol detection kit (CHOL, Roche/Hitachi, Roche Diagnostics).

### Analysis of gene expression by real-time PCR

A Nucleospin RNA II kit (Macherey-Nagel) was used for the RNA extraction, and a high capacity cDNA synthesis kit (Applied Biosystems) was used to perform the cDNA synthesis. Quantitative real-time PCR was performed with a 7900HT PCR machine (Applied Biosystems), FastStart Universal Probe Master ROX (Roche), and a FAM-labeled TaqMan primer and probe for mouse *BCMO1* (316000, Roche). We used *Gapdh* (307884, Roche) as a reference gene.

### Statistical analyses

The student t-test was used to compare the different treatments with the control treatment. Significance was considered to be p<0.05, and the values in the text are the means ± SE.

## Results

### Dietary all-trans and 9-cis β-carotene accumulation in mouse macrophages *in-vivo*

First, we investigated whether all-trans and 9-cis β-carotene isomers, provided in the mouse feed, are accumulated in macrophages *in-vivo*. Following a 6 week enrichment of a high-fat diet with carotenoids, provided as *Dunaliella* powder, we detected higher levels of both all-trans and 9-cis β-carotene in the peritoneal macrophages isolated from the LDLR-/- mice treated with *Dunaliella,* compared to the control mice. Both isomers were also detected in the plasma of non-fasting animals fed *Dunaliella* powder, while no carotenoids were detected in the plasma of the control mice (Table 1).

**Table 1:** The β-carotene content in mouse plasma and in isolated peritoneal macrophages.

	**β-carotene(μg per mL plasma)**	**β-carotene(ng per 10^6^ cells)**
**Plasma Control**	0	-
**Plasma *Dunaliella***	0.266	-
φ* Control	-	0.043
**φ *Dunaliella***	-	0.064

* φ = macrophages

Carotenoids were extracted following 6 weeks of a western diet with or without *Dunaliella* enrichment. Carotenoids were extracted from a pool of plasma (n = 5 mice) and a pool of macrophages (n = 5 mice). The assay was repeated twice and representative results are presented.

### 9-cis β-carotene and its metabolites inhibited foam cell formation *in-vitro*

**Figure 1:**
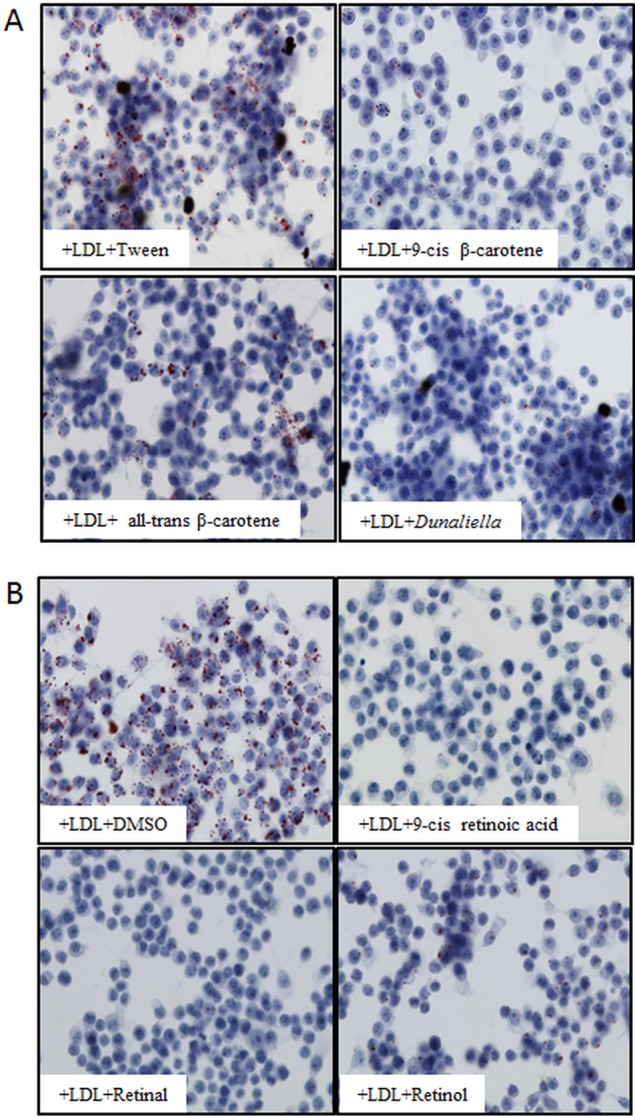
9-*cis* β-carotene and its metabolites inhibited foam cell formation *in-vitro*. Oil Red O staining of Raw264.7 cells loaded with minimally modified LDL. 10μM of 9-cis β-carotene, all-trans β-carotene or *Dunaliella* extract with Tween served as a control (A). 5μM of 9-cis retinoic acid, retinol or retinal with DMSO serves as a control (B). Lipid droplets colored in red.

The effects of 9-cis β-carotene on foam cell formation were first investigated *in-vitro* in the Raw264.7 macrophage cell line. To obtain foam cells, the macrophages were incubated with minimally modified LDL (mmLDL) for 24 hours; 9-cis β-carotene and *Dunaliella* extract significantly inhibited foam cell formation, as indicated by the reduced number of oil-red stained globules (Fig. 1A). Next, we investigated whether β-carotene metabolites inhibit this process, and found that similar to β-carotene, retinol, retinal and 9-cis retinoic acid also inhibited foam cell formation (Fig. 1B).

### 9-cis β-carotene enriched diet inhibited foam cell formation *ex-vivo* and *in-vivo*

**Figure 2:**
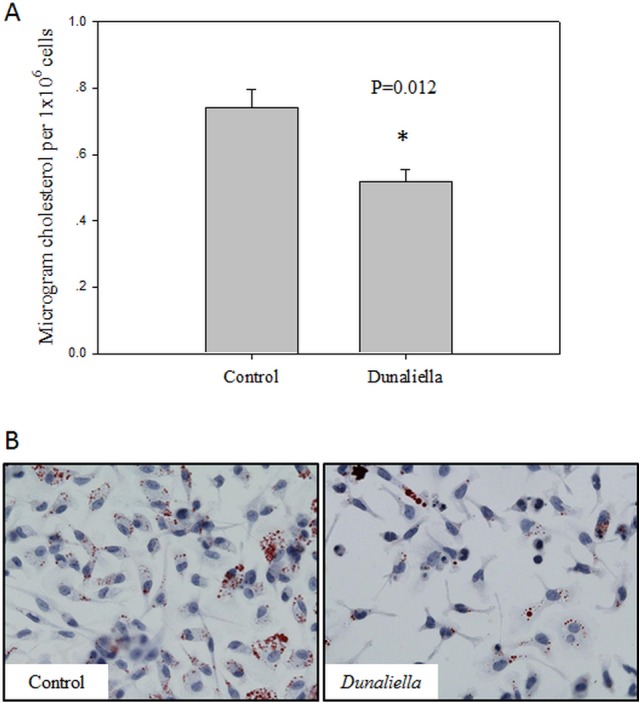
9-cis β-carotene enriched diet inhibited foam cell formation *ex-vivo.* Cholesterol content in the cells was measured after Folch extraction and colorimetric reaction. Values are means ±SE, n = 5. * *P*<0.05 as measured by student t-test (A). One representative picture of Oil Red O staining of macrophages isolated from one mouse in each group (B). Lipid droplets colored in red.

**Figure 3:**
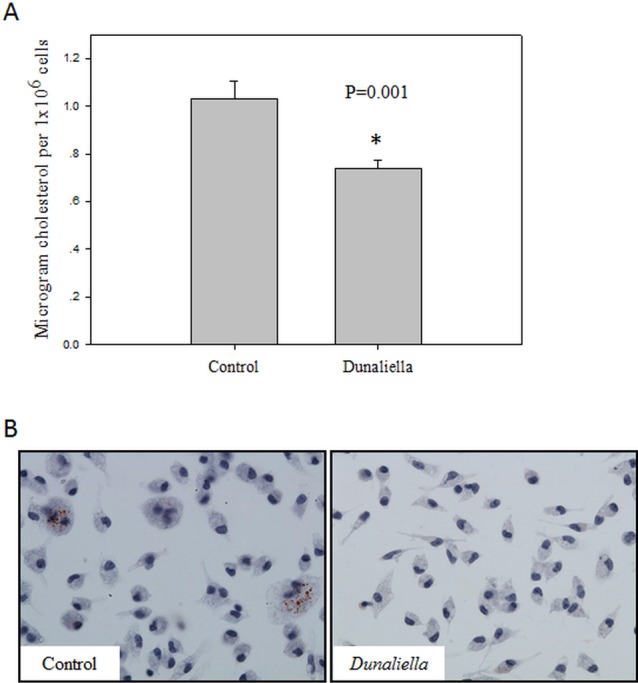
9-cis β-carotene enriched diet inhibited foam cell formation *in-vivo.* Cholesterol content in the cells was measured after Folch extraction and colorimetric reaction. Values are means ±SE, n = 5. * *P*<0.05 as measured by student t-test (A). One representative picture of Oil Red O staining of macrophages isolated from one mouse in each group (B). Lipid droplets colored in red.

In order to study the effects of dietary β-carotene on foam cell formation in mice, LDLR-/- mice were fed a chow diet enriched with *Dunaliella* powder, containing high levels of all-trans and 9-cis β-carotene. Peritoneal macrophages were isolated and incubated with mmLDL for 24 hours. We found that dietary treatment with *Dunaliella* reduced the number of oily globules and significantly lowered the cellular cholesterol content in *Dunaliella*-treated mice, compared to the controls (Fig. 2A, B). To induce foam cell formation *in-vivo*, LDLR-/- mice were fed a high-fat high-cholesterol western diet enriched with *Dunaliella* for 6 weeks. Similar to the *ex-vivo* results, less cholesterol accumulated in the peritoneal macrophages isolated from *Dunaliella*-treated mice compared to the control, untreated mice (Fig. 3A, B).

### BCMO1 is expressed and active in macrophages

**Figure 4:**
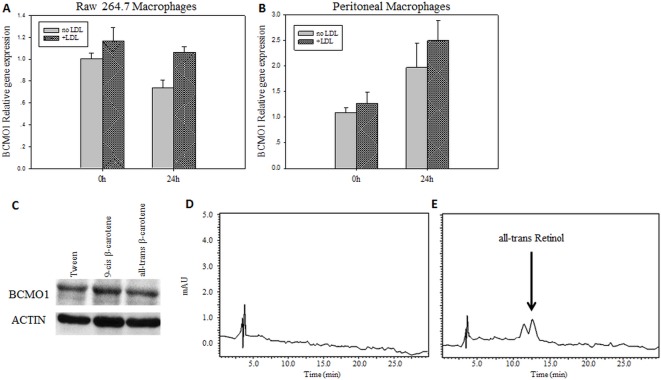
BCMO1 is expressed and active in macrophages. BCMO1 mRNA expression levels as measured by real time PCR and normalized to endogenous gene, Gapdh. Values are means ±SE and normalized to fold change in untreated cells (no LDL). Raw264.7 cells (A) and peritoneal macrophages isolated from LDLR-/- mice (B). BCMO1 protein as determined by western blot analysis. The results represent one of three independent experiments (C). HPLC chromatogram shows retinol production in Raw264.7 cells after 24-hour incubation with 9-cis β-carotene. Total retinol content after control treatment (D) and after 9-cis β-carotene incubation (E). Separation was conducted on C18 column and detection at 325 nm.

We hypothesized that β-carotene can be converted to retinoids in macrophages. Therefore, we first tested whether BCMO1 (β-carotene cleavage enzyme) is expressed and active in macrophages. By using real-time PCR, we found that BCMO1 is expressed in both the Raw264.7 cell line and primary mouse peritoneal macrophages, with similar expression levels, in their regular or fat-loaded forms (Fig. 4A, B). Western blot analysis showed that BCMO1 protein is expressed in Raw264.7 macrophages and β-carotene isomers had no effect on BCMO1 protein levels (Fig. 4C). Moreover, when using 9-cis β-carotene or *Dunaliella* extract as substrates, we found that β-carotene is converted to retinol in these cells (Fig. 4D, E, Table 2), suggesting that BCMO1 is active in these cells.

**Table 2:** Retinol content in Raw264.7 cells following β-carotene treatments.

**Treatment**	**Retinol(ng per 10^6^ cell seeded)**
Tween	0
9-cis β-carotene	0.8±0.53*
*Dunaliella*	2.31±1.56*

Retinol content as measured after HPLC separation and 325nm detection. Content set by standard curve of known concentrations (n = 3).

* p<0.05 compared to control.

### 9-cis β-carotene activated RXR in cell culture

**Figure 5:**
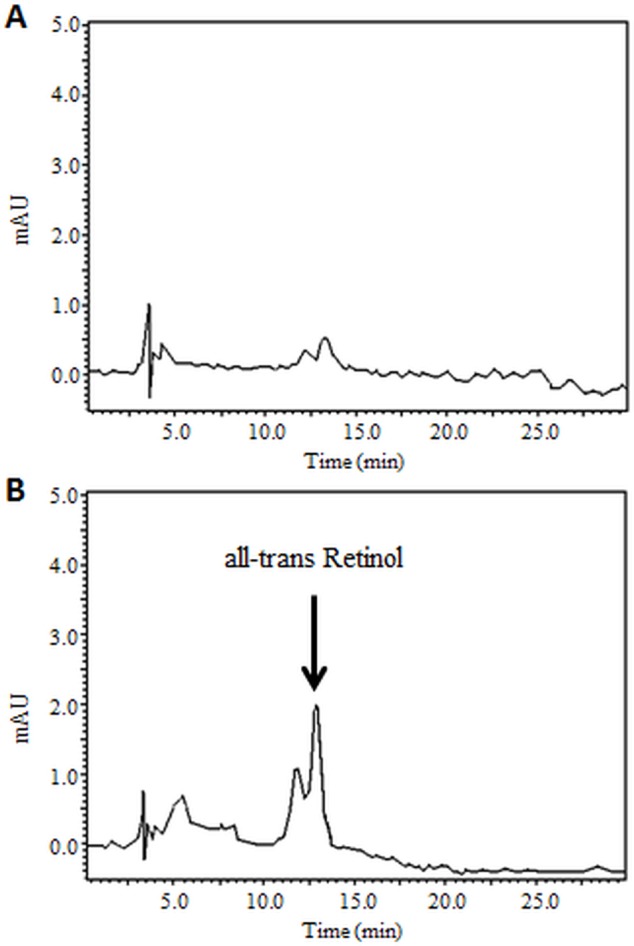
Retinol production in Hepa1-6 cells. HPLC chromatogram shows retinol production in Hepa1-6 cells after 24-hour incubation with 9-cis β-carotene. Total retinol content after control treatment (A) and after 9-cis β-carotene incubation (B). Separation was conducted on C18 column and detection at 325 nm.

**Figure 6:**
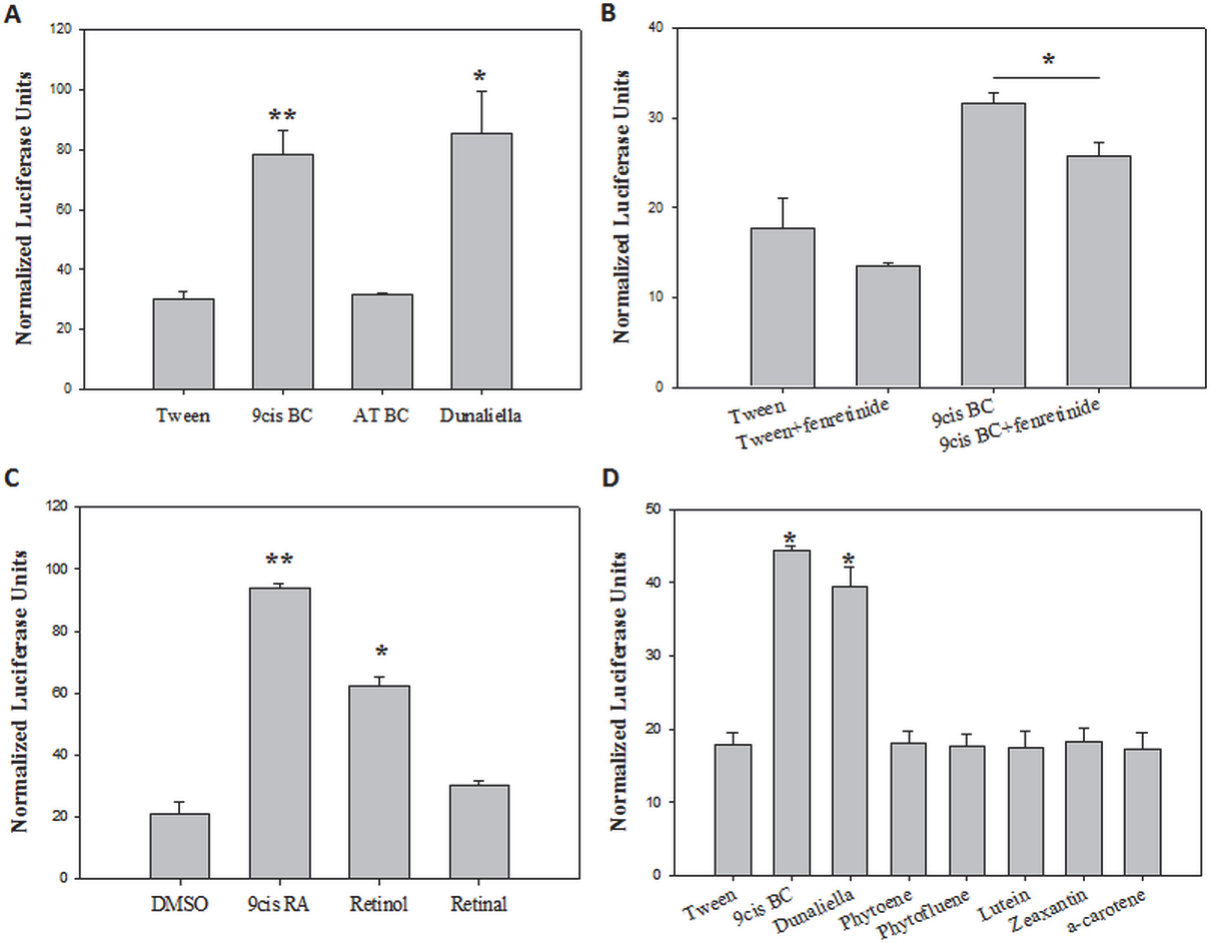
RXR activation in Hepa1-6 cells. Luciferase activity as measured in Hepa1-6 cells and normalized to transfection efficiency by *Renilla*. 9-cis β-carotene and *Dunaliella* activate RXR (A). Fenretinide (BCMO1 inhibitor) inhibited RXR activation by 9-cis β-carotene (B). 9-cis retinoic acid and retinol activate RXR (C). Carotenoids from the alga other then 9-cis β-carotene did not activate RXR (D). Tween and DMSO served as control treatments. Values are means ±SE, n = 2–4. * *P*<0.05, ** *P*<0.01 as measured by student t-test.

According to our working hypothesis, 9-cis β-carotene is converted to retinoids and activates nuclear receptors; therefore, we sought to study the effects of 9-cis β-carotene on the activation of the nuclear receptor RXR. We have used Hepa1-6 cells where BCMO1 expression was previously shown [33]. Incubation of these cells with 9-cis β-carotene resulted in the accumulation of all-trans retinol (Fig. 5A, B). Much lower levels of retinol were detected in the untreated cells. Interestingly, retinol was not formed following incubation with synthetic all-trans β-carotene (Table 3). The effects of 9-cis β-carotene on RXR activity were studied by transfection of the cells with the RXR-luciferase reporter plasmid; 9-cis β-carotene and *Dunaliella* extract similarly activated RXR, while all-trans β-carotene did not increase the RXR activity above basal levels (Fig. 6A). To assess whether the activation of RXR is BCMO1-dependent, we incubated the cells in the presence of a BCMO1 inhibitor, fenretinide [34]. A partial, although significant inhibition of luciferase activity was observed, indicating that at least part of the RXR activation is dependent on BCMO1 activity (Fig. 6B). Next, we assessed the effects of β-carotene metabolites on RXR activity. While both retinol and 9-cis retinoic acid increased activity significantly, the retinal did not (Fig. 6C).

**Table 3:** Retinol content in Hepa1–6 cells following β-carotene treatments.

**Treatment**	**Retinol(ng per 10^6^ cell seeded)**
Tween	0.38±0.34
9-cis β-carotene	1.81±0.93*
all-trans β-carotene	0.63±0.23
*Dunaliella*	3.96±0.76*

Retinol content as measured after HPLC separation and 325 nm detection. Content set by standard curve of known concentrations (n = 3).

* p<0.05 compared to control.

Finally, we examined whether other carotenoids present in *Dunaliella* can activate RXR. We incubated the cells in the presence of the β-carotene precursor’s phytoene and phytofluene, with the xanthophylls lutein and zeaxanthin, and with the pro-vitamin A precursor, α-carotene. None of the tested substances activated RXR like the 9-cis β-carotene or the *Dunaliella* extract (Fig. 6D).

## Discussion

In this study, we demonstrated that dietary fortification with the alga *Dunaliella* leads to 9-cis and all-trans β-carotene accumulation in peritoneal macrophages, and that 9-cis β-carotene and *Dunaliella* lipid extract inhibited foam cell formation. We also showed, for the first time, that BCMO1 is expressed and active in macrophages, suggesting that the local conversion of β-carotene to retinoids can take place in macrophages and consequently mediate the inhibition of foam cell formation.

First, we showed that food fortification with the algal powder of *Dunaliella* leads to 9-cis and all-trans β-carotene accumulation in mouse peritoneal macrophages. We and others have previously shown that feed fortification with the alga *Dunaliella* increases β-carotene accumulation in the liver of animal models [15,35]. In addition, the consumption of a carotenoid rich diet increases the β-carotene levels in the blood serum and spleen [36], as well as in the lungs and kidneys [37]; but to the best of our knowledge, no one analyzed carotenoid accumulation in macrophages.

Previous results in our laboratory showed that food fortification with the 9-cis β-carotene rich alga *Dunaliella bardawil* inhibits atherosclerosis development in LDLR-/- [15] and apoE-/- mice [16]. We, therefore, sought to study the effects of 9-cis β-carotene and other retinoid cleavage products on macrophage foam cell formation, the hallmark of early atherosclerotic lesions. The 9-cis β-carotene and *Dunaliella* lipid extract inhibited foam cell formation, similar to 9-cis retinoic acid, while the retinol and retinal only partially inhibited this process. Further, we found that a diet enriched with the alga inhibited foam cell formation; *ex-vivo,* in isolated peritoneal macrophages, and *in-vivo*, in mice fed an atherogenic diet.

Previous studies have examined the effects of carotenoids and retinoids on macrophage foam cell formation. Nagai et al. found that Am80, a synthetic retinoid and RAR agonist, inhibits foam cell formation in peritoneal macrophages [38]. Dushkin found that 9-cis retinoic acid inhibits foam cell formation *in-vivo* and reduced cholesterol, CE and TG accumulation in these cells [39]. Bravo, in her work, found that the carotenoid lycopene significantly inhibited cholesterol esterification during foam cell development in human monocyte-derived macrophages [21]. Recently, it was shown that all-trans retinoic acid, retinol and all-trans β-carotene inhibited macrophage phagocytosis [40]. In addition to cholesterol and fat accumulation, macrophages serve as important regulators of inflammation in atherosclerosis development; while β-carotene induced changes in the inflammatory response in the Raw264.7 macrophage cell line [41] and a reduction in IL-6 and TNF-α secretion to the medium [42]. These works are in accord with our results, and show that carotenoids and retinoids have a wide impact on macrophage properties *in-vivo* and *in-vitro*.

9-cis β-carotene is a precursor for 9-cis retinoic-acid, the nuclear receptor RXR natural ligand. Thus, we next examined whether the alga carotenoids activate RXR. We established a cellular system using Hepa1-6 cells (murine hepatic cells), in which we have demonstrated the activity of the BCMO1 enzyme. We found that 9-cis β-carotene and *Dunaliella* lipid extract activate RXR in the Hepa1-6 cell-line. Recent work has shown that all-trans retinoic-acid, as well as all-trans β-carotene, activated the nuclear receptor RAR in the Raw264.7 cell line [40]. However, it is not clear whether β-carotene activated RAR directly or by its transformation to retinoids. This was investigated by Park et al. who examined RAR activation by β-carotene, mediated by BCMO1 activity. Unlike our research, where the endogenic activity of BCMO1 in Hepa1-6 cells was assayed; in that work, the cells were transfected with a BCMO1 plasmid and showed an increase is RAR activity, along with an elevation in β-carotene concentration [43].

Dietary enrichment with *Dunaliella* led to carotenoid accumulation in macrophages, as well as the inhibition of foam cell formation. These finding led us to examine whether the β-carotene cleavage enzyme, BCMO1, is both expressed and active in macrophages. We found that BCMO1 mRNA is similarly expressed in native macrophages as well as in foam cells. We also showed that BCMO1 protein is present in the macrophages. Moreover, BCMO1 is active and can produce retinol from 9-cis β-carotene administrated to macrophages in the cell culture. In order to investigate whether RXR activation by 9-cis β-carotene is BCMO1 dependent, we inhibited the BCMO1 enzyme by fenretinide and found that this treatment partially inhibited the RXR activation, suggesting that 9-cis β-carotene activates RXR in this system by its conversion to retinoids. However, it seems that there are bypass tracks for β-carotene cleavage, other than BCMO1, such as the additional cleavage enzyme BCDO2 which is expressed in these cells (data not shown).

It is important to note that the *in-vivo* experiment which examined the effect of 9-cis β-carotene on atherogenesis in LDLR-/- mice found that *Dunaliella* inhibited atherosclerosis development more significantly than treatment with the isolated 9-cis β-carotene isomer. It seems that additional ingredients in the alga in combination with the alga carotenoids inhibit atherosclerosis development more efficiently than isolated 9-cis β-carotene. We, therefore, tested whether other carotenoids of the alga activate RXR. We found that other carotenoids in the alga, such as α-carotene, luteine, zeaxantin, phytoene and phytofluene, did not activate RXR. It turned out that among the alga carotenoids that have been tested, only 9cis βc activated the nuclear receptor RXR.

The results presented in this study support the hypothesis that 9-cis β-carotene activates RXR by forming vitamin A and 9-cis retinoic-acid. We examined RXR activation in hepatocytes, an important site of vitamin A metabolism and in atherosclerosis development [44]. The β-carotene enriched diet resulted in β-carotene accumulation in several tissues, such as the uterus, testes and lungs [45] as well as the blood serum and spleen [36]. Moreover, BCMO1 expression was demonstrated in the stomach, bowel, pancreas, testes, ovaries, kidney, skeletal muscle and retinal cells [46–48]. Therefore, carotenoids from the diet, transported by chylomicrons, can reach a wide range of tissues and can be converted locally to 9-cis retinoic acid and other retinoids, and potentially activate the nuclear receptors RXR and RAR.

In conclusion, carotenoids (including 9-cis β-carotene) transported by lipoproteins can reach a wide range of tissues and, as demonstrated for the first time in this work, accumulate in macrophages. Moreover, this work is the first to show the expression and activity of the β-carotene cleavage enzyme BCMO1 in macrophages. In addition, we demonstrated that 9-cis β-carotene activates the nuclear receptor RXR. Thus, the results support the hypothesis that β-carotene can accumulate in the macrophages, be cleaved by endogenous BCMO1 to form 9-cis retinoic acid and other retinoids and, subsequently, activate the nuclear receptor RXR. RXR, along with additional nuclear receptors, can affect different metabolic pathways, including the development of macrophage foam cells and atherosclerosis.
